# The Treatment of Empyema by Incision and Drainage, as Illustrated by Forty Cases

**Published:** 1886-12

**Authors:** 

**Affiliations:** House Surgeon Bristol General Hospital


### THE TREATMENT OF EMPYEMA BY INCISION AND DRAINAGE, AS ILLUSTRATED BY FORTY CASES.

BY

DR. NEWNHAM,

*House Surgeon Bristol General Hospital.*

My chief object in bringing forward such a hackneyed subject, is to emphasise the necessity of free drainage in these cases. In my humble opinion, aspiration should only be used as a means of preliminary diagnosis; for I feel very doubtful if we could effect a complete cure by it alone. Also I would raise the point, "Is it necessary to excise a portion of rib or ribs?"—a practice which is universally adopted at some hospitals now. My present idea, from the results of the cases I am quoting from, would lead me to say that I believe it is not necessary, and I think it adds somewhat to the severity of the operation: still, this is only my opinion; and it has been maintained that in children it is a necessity. In thirty-nine cases occurring in young subjects under the age of twelve years, it was not found necessary to remove any portion of any rib. I was always able to insert my tube between the ribs, and the statistics themselves prove that the results are as favourable as anyone could possibly expect to get.

Why is it that we meet with such a large number of cases of empyema amongst the children of the poor, while it is such a very rare affection in those of the wealthy classes ? I would suggest that they are simply untreated cases of pleurisy, to begin with; and the children being allowed to run about, being badly fed, and living in a bad atmosphere, the fibrino-serous effusion thus has a tendency to become purulent: such cases amongst the wealthier classes, on the other hand, under proper treatment, would probably remain simple effusions, and finally clear up altogether. So, if we state the matter broadly, we can say that it is all accounted for by bad hygienic conditions ; for we know that an effusion is never purulent at the commencement, but always fibrino-serous—this has been experimentally proved.

At one time it was asserted that empyema never occurred in children under the age of six years. Ziemssen was one of the first to refute this ; for he collected statistics of his younger cases, and showed that seventeen out of fifty-four occurred under six years of age, but of my *forty* cases *thirty-six* have been in children under six years. The reason for this statement is obvious : the medical attendant often fails to examine the chest of a child ; whereas, if he would only look at the case from the same point of view as he would his adult patient, he would find no difficulty at all, and we should be spared such very rash statements.

Another question that may occur to us is, " How is it that such enormous quantities of pus are again and again effused ?" May it be that a rapid process of cell division amongst the colourless cells, which have wandered out of their vessels and are present in the fibrino-serous effusion, takes place, and so an exudation which was at first fibrino-serous becomes purulent ?

The tube which I have used consists of an ordinary piece of drainage tube threaded through a circle of Martin's bandage, then divided into four pieces and tacked down with wire at four separate spots. I do not claim any originality for it; but I think it is very simple, easily made, and accessible to all.

The case on which I founded my paper was a boy, aged eleven years, admitted into the Bristol General Hospital on January 15th, 1886, under the care of Mr. Keall. The right chest was full of pus, and three days after admission the chest was incised in the eighth space, with all antiseptic precautions, and the tube introduced. As usual, a very large quantity of thick curdy pus escaped, which it would have been utterly impossible to get out by means of an aspiration. This boy did remarkably well: the antiseptic dressings were changed daily at first, and the tube taken out, washed and replaced; and on February 8th (twenty-one days after the operation) the tube was left out altogether, and a week subsequently the boy was discharged cured. The tube had to be kept in the chest rather longer that in some of my cases; but this boy had been ill for three weeks before he was seen by a medical man. I venture to think that if we could only get the cases under treatment sooner, our results would be still better.

Of the forty cases of empyema I have treated myself, one died; this was a child of five years old, admitted too late for any treatment to be of use, for the pus had perforated the diaphragm and set up peritonitis. Thirty-six were discharged from the hospital quite well: four of these healed fourteen days after the operation, and the other thirty-two at variable times, not exceeding eight weeks. Three were sent out with a small sinus still left; there was a very small amount of discharge, and they had been under treatment for eight weeks.

The statistics of these cases have been already published by Dr. Goodhart, in his work on *The Diseases of Children*. They all occurred at the Evelina Hospital for Sick Children.

In the last nine months we have had four cases, in which I have incised the chest for empyema :

1. A child under the care of Dr. Baron. Here the result was perfect ; it was well and left the hospital four weeks after the operation.

2. A girl, aged 24, also under the care of Dr. Baron. Now, this case had been aspirated three times at the Infirmary, and she came on to the Hospital, as patients often do ; they try first one place and then another. On the day of her admission I incised the chest, let out about ten ounces of pus ; and in ten days I was able to take out the tube, and the wound healed up within a week.

3. A girl, aged 22, under the care of Dr. Markham Skerritt. She was admitted with the left chest full of pus, the heart's apex being felt just under the right nipple. In this case also the chest was incised directly, and although the patient was almost moribund, she made a good recovery. The wound quite healed, and she left the hospital in six weeks after the operation.

4. A man, aged 26, with the left chest full of pus. This is still under treatment, having only been operated on a week ago.

I may say here, that I never wash the chest out, or syringe any lotion into it of any kind or description, and for this reason : cases have occurred in which the patient has suddenly fallen back in bed and died. Therefore, I

think it is a very dangerous practice; and I also believe, from my small experience, it is quite unnecessary.

An argument is occasionally brought forward by some surgeons, that in the adult you will *always* have a sinus left. My answer to this would be, that if the case is taken sufficiently early and treated properly, the operator will never have cause to regret it, and he will never get a sinus left; out of forty-five now, I have had only three with a sinus, and it is only fair to assume that as they did not appear again, those cases also must have healed.

I will now quote one or two cases more in detail:

1. G. H., girl aged 4½ years. Admitted August 4th, 1884, to the Evelina Hospital. Left chest dull from apex to base; breath sounds distant all over, but not absent at any one point. Circumference of chest showed the right to be nine inches, and the left nine and a half inches. T. 98.6, P. 120. Incised the same day; large tube inserted after the pus had escaped. Dressed with all antiseptic precautions. The wound had healed, and the child was quite well on August 26th, or *twenty-two* days after the operation.

2. F. D., boy aged 4 years. Admitted on August 31st, 1884. Right chest full of pus; patient very livid and breathless. T. 98.4, P. 140. As the father objected to the boy taking chloroform, I did the operation as quickly as I could without. The case did well; the wound closed, and the boy was discharged on October 2nd, or *thirty-three* days after the operation.

3. M. R., girl aged 4 years. Admitted with the left chest full of pus. Operated on the same day, January 3rd, 1885; fifteen ounces of pus escaped. Dressed in exactly the same way as all the others, and on January

17th, or *fourteen* days after the operation, the wound had quite healed.

4. L. L., girl aged 4½ years. Right chest full of pus. Sent to me by a medical man, who requested me for charity to give the child a place to die in. The operation was done the same day; over forty ounces of pus were measured. This case also did well; but the wound had not quite healed until the forty-sixth day after the operation.

5. E. J., girl aged 6 years. Left chest full of pus. Wound had quite healed thirty days after the operation.

6. H. H., a boy aged 7 years. Empyema on right side. In this case I tried a free incision, without inserting any tube. As may be imagined, it was not a success; the wound closed on the surface, and two days later I had to re-open it and put in a drainage tube. The boy was not well until the thirty-fourth day after the operation.

It would be of little interest for me thus to quote the whole forty cases; I will merely mention the fact, that I could give some of the results of repeated aspirations which it has been my misfortune to meet with, but I will refrain.

